# Open-source analysis and visualization of segmented vasculature datasets with VesselVio

**DOI:** 10.1016/j.crmeth.2022.100189

**Published:** 2022-03-23

**Authors:** Jacob R. Bumgarner, Randy J. Nelson

**Affiliations:** 1 Department of Neuroscience, Rockefeller Neuroscience Institute, West Virginia University, Morgantown, WV 26505, USA

**Keywords:** vasculature, vasculature analysis, vasculature visualization, cerebrovasculature, open-source application, Python, vascular networks

## Abstract

Vascular networks are fundamental components of biological systems. Quantitative analysis and observation of the features of these networks can improve our understanding of their roles in health and disease. Recent advancements in imaging technologies have enabled the generation of large-scale vasculature datasets, but barriers to analyzing these datasets remain. Modern analysis options are mainly limited to paid applications or open-source terminal-based software that requires programming knowledge with high learning curves. Here, we describe VesselVio, an open-source application developed to analyze and visualize pre-binarized vasculature datasets and pre-constructed vascular graphs. Vasculature datasets and graphs can be loaded with annotations and processed with custom parameters. Here, the program is tested on ground-truth datasets and is compared with current pipelines. The utility of VesselVio is demonstrated by the analysis of multiple formats of 2D and 3D datasets acquired with several imaging modalities, including annotated mouse whole-brain vasculature volumes.

## Highlights

• VesselVio is an application created to analyze segmented vasculature datasets
• Custom analysis pipelines provide detailed network and segment features
• Interactive dataset visualization enables result inspection and image creation
• VesselVio can be freely downloaded or run from the terminal with a single line of code

## Motivation

Modern imaging techniques and open-source image segmentation packages have led to the generation of large-scale and high-resolution vasculature datasets. However, open-source analysis packages for these datasets require programming knowledge, leading to an access barrier for some researchers. To overcome this barrier, we present VesselVio, a standalone and open-source application developed for the analysis and visualization of vasculature datasets.

## Graphical abstract

**Figure:**
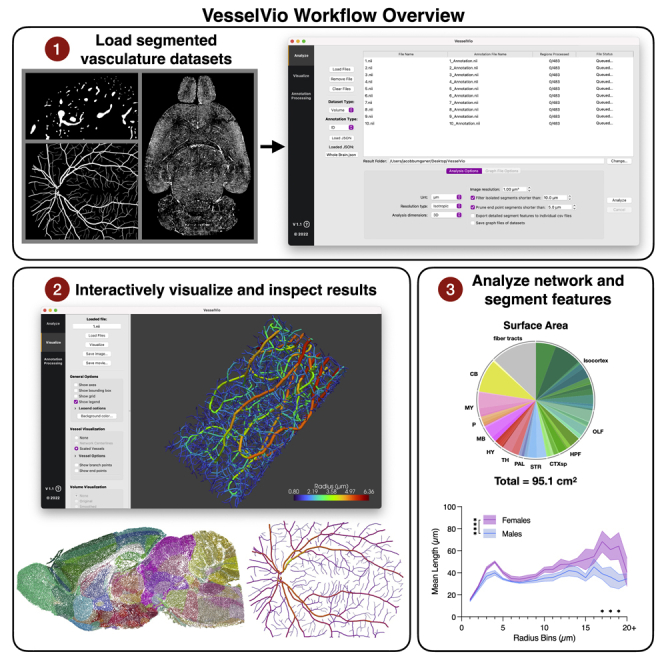

## Teaser

Bumgarner et al. present an open-source application, named VesselVio, that was designed to facilitate the process of vasculature dataset analysis. Their application provides an accessible method for researchers to extract and visualize detailed features from numerous formats of vasculature datasets, including annotated mouse-brain volumes.

## Introduction

The acquisition of high-resolution and large-scale 3D vasculature datasets has been facilitated by recent and continual developments of powerful imaging technologies, including light-sheet fluorescence microscopy (LSM) (Di Giovanna et al., 2018; Kirst et al., 2020; Todorov et al., 2020) and micro-computed tomography (μCT) (Quintana et al., 2019; Schaad et al., 2017). Simultaneously, constant improvements in computational power and the widespread availability of powerful programming languages provide great means to process and extract detailed features from the resulting datasets. Use of micron-scaled imaging technologies and powerful computational tools to characterize the intricate details of microvascular networks will improve understanding of microvascular structure, function, and remodeling in health and disease.

Despite ongoing advancements in imaging technologies and open-source image segmentation software for 3D vasculature datasets (Kirst et al., 2020; Todorov et al., 2020; Haft-Javaherian et al., 2019), the publication of open-source and stand-alone analysis software applications that do not require prior programming experience is scarce. Existing standalone analysis tools that are accessible and freely available are unable to extract features from 3D datasets (Zudaire et al., 2011; Niemisto et al., 2005). Other current open-access, terminal-based analysis packages have similar 2D limitations (Mazzaferri et al., 2018; Montoya-Zegarra et al., 2019; Rust et al., 2020). Open-source analysis tools and software packages capable of analyzing 3D datasets are limited. Several of these tools depend on uncorrected centerline analyses, provide limited feature output, and markedly over-label branch points (Arganda-Carreras et al., 2010; Todorov et al., 2020). Other modern analysis packages that extract more accurate (Kirst et al., 2020; Tetteh et al., 2020) and detailed features (Hahn et al., 2019; Chapman et al., 2015) from 3D vascular networks require considerable programming skills or heavy interaction with terminals, potentially leading to unwelcome usage barriers and steep learning curves for researchers. Many publications also make use of private code or proprietary software for feature extractions (Jafarnejad et al., 2019; Epah et al., 2018; Kelch et al., 2015). In these instances, limited code availability and high software prices can hinder widespread analysis access. Thus, there is an apparent need for an easily accessible tool for 3D vasculature-dataset analysis.

Here, we present *VesselVio*, an open-source application for the analysis and visualization of vasculature datasets and vascular graphs. The back-end pipelines leverage custom feature-extraction techniques, high-level Python libraries, just-in-time compilation, and parallel processing for rapid, detailed feature extraction and visualization of vasculature datasets. To make these analysis pipelines easily accessible, we also developed a downloadable (or, alternatively, single-line executable) front-end application. To test the performance and utility of VesselVio, we analyzed ground-truth synthetic vascular datasets, annotated mouse whole-brain datasets imaged using LSM, mouse inferior colliculus segmentations imaged using μCT, 2D retinography images, and pre-constructed mouse whole-brain vascular graphs.

## Results

### Graphical user interface enables dataset loading, analysis, and visualization

**Figure 1:**
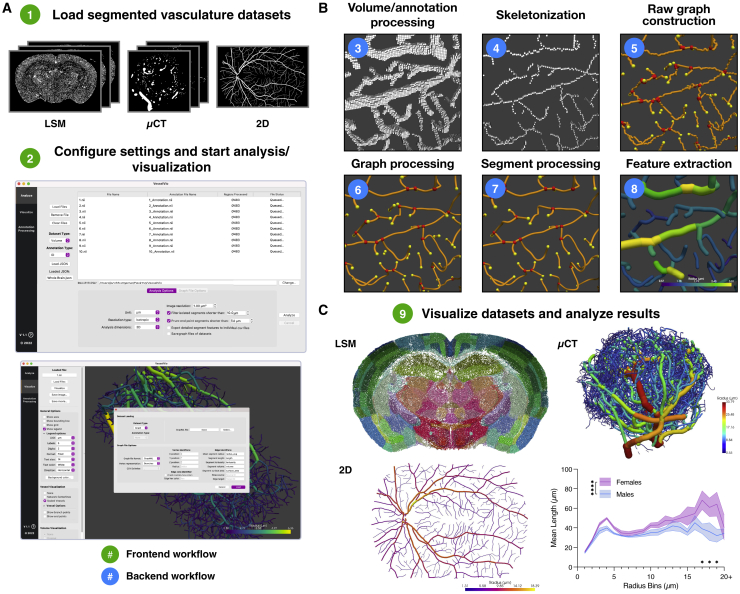
Overview of the VesselVio pipeline for vascular network processing (A) Pre-binarized datasets, annotated volumes, and graphs are loaded into the program with custom analysis and feature export parameters. (B) Centerlines are extracted from the loaded datasets to create undirected graphs. Centerlines are smoothed, spurious branchpoints are filtered, and segments are processed prior to feature extraction. (C) The resulting datasets can be visualized for inspection of result accuracy with subsequent result export for analysis.

We sought to build an open-source application that allows users to extract and visualize numerous quantitative features from vascular networks (Figures S1A–S1C). VesselVio was developed for vascular datasets that have already been binarized, making it compatible with datasets of any imaging origin (Figure 1A). The application is also compatible with pre-constructed graphs from other programs. Further, binarized volumes can be loaded alongside annotations, such as the p56 mouse brain atlas from the Allen Brain Institute, to analyze region-specific features (Figure 1C).

### Centerline extraction and graph construction enable detailed network feature extraction

The features of a vascular network can be extracted by identifying centerlines of the network and creating undirected graphs from the centerline points (Selle et al., 2002; Czech et al., 2011; Lee et al., 1994). Local neighborhood connectivity of the skeleton points is identified and used to create an undirected graph, G = (V, E). Graphs are constructed using the Python package igraph, which was selected because of its efficiency with graph loading and shortest-path finding, as well as its native compatibility with Windows and MacOS. Following initial graph construction, the datasets undergo a series of correction processes that eliminate spurious branchpoints, smooth centerlines, and remove isolated/endpoint segments at user-defined lengths (Figure 1B). These initial processing stages enable downstream quantifications of network and segment features (Figure 1C).

### Spurious branchpoint clique filtering improves branchpoint quantification

**Figure 2:**
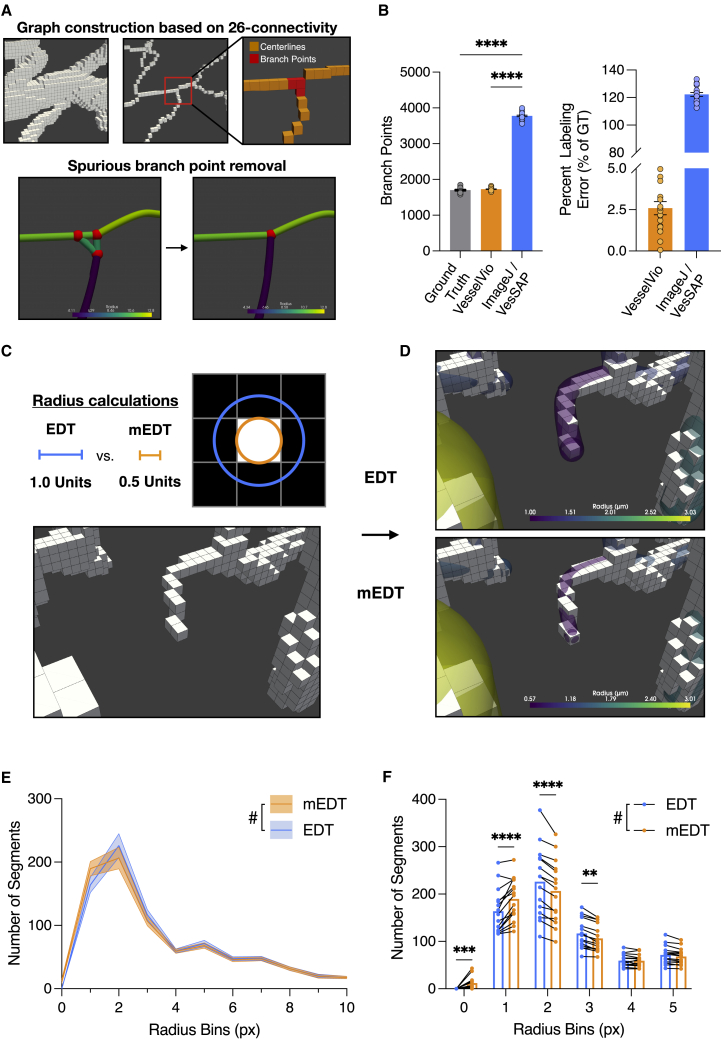
Spurious branchpoint filtering and modified radius calculations improve feature quantification (A) Clique clusters formed by spuriously labeled branchpoints are eliminated by weighting the radius of the candidate points and their neighbors. (B) Branchpoint filtering compared with ground-truth-labeled synthetic vasculature datasets and an existing analysis pipeline (n = 20). (C) Modified Euclidean distance transform (mEDT) radius calculations for non-vessel neighbors connected to voxel faces or pixel edges. (D) Comparisons between EDT and mEDT radius calculations for an edge vessel with single-voxel width at numerous points. (E) Comparison of the distribution of segments along 0–10 px radius bins from the glaucomatous HRF images (n = 15). (F) Repeated measures analysis of the change of radii in 0–5 px radius bins using EDT and mEDT. Data are represented as mean ± SEM. (B) Data analyzed using a one-way ANOVA. (E and F) Data analyzed using a repeated measures two-way ANOVA. Multiple comparisons conducted using Sidak’s test. #, interaction of measurement method × segment size. ∗∗p < 0.01, ∗∗∗p < 0.001, ∗∗∗∗p < 0.0001.

After graph construction, network endpoints and branchpoints are identified by examining the degree of connectivity of the centerline vertices (Figure 2A), a commonly used approach (Arganda-Carreras et al., 2010; Hahn et al., 2019; Todorov et al., 2020). Although centerline neighbor 26-connectivity can be used to accurately detect endpoints, this approach spuriously over-labels branch points, leading to artificially inflated counts (Figure 2B). Some programs allow for interactive user input to correct mislabeled segments, inaccurate centerlines, or spurious branchpoints (e.g., Imaris [Quintana et al., 2019]). However, manually correcting branchpoints in 3D datasets can become tedious and time intensive, particularly when datasets are many gigabytes large. Presented here is an automated approach to correct these spuriously labeled branchpoints.

Spuriously labeled branchpoints form small, all-to-all connected subgraphs in the constructed graphs, otherwise known as cliques. Previous techniques used to eliminate spurious branchpoint cliques rely on parent vessel orientations (Palágyi et al., 2006) or generate candidate weights based on 26-neighborhood connectivity (Chen et al., 2009). VesselVio implements a two-pass filter that eliminates spurious branchpoint cliques through weighting based on the candidate vertex radius and the radii of neighboring vertices. Weighting candidates based on radius in addition to connectivity also mimics parent vessel hierarchy and improves segment radii calculations (Figure 2A).

To test our approach, a set of synthetically generated vasculature datasets with ground-truth branchpoint labels were acquired and analyzed (Tetteh et al., 2020), and results were compared with previous programs (Arganda-Carreras et al., 2010; Todorov et al., 2020). VesselVio clique filtering resulted in a mean 97.4% accuracy of branchpoint labeling (n = 20). Further, the hierarchical branchpoint filtering of VesselVio results in a mean 2.6% error that outperforms the mean 122.3% error of previous techniques (Figure 2B).

### Modified Euclidean distance calculations for segment radius estimation

Several techniques exist for identifying vessel radii. One technique involves recording the largest maximally inscribed spheres that can rest within mesh vessel centerline points (Antiga et al., 2003; Antiga and Steinman, 2004), but this technique often depends on the creation of directed graphs (i.e., manually directed vessel hierarchy) and thus was not suitable for an automated pipeline. A similar method identifies the Euclidian distance between a vessel centerline and the center of the nearest non-vessel neighbor (Kirst et al., 2020; Mouches and Forkert, 2019). However, one pitfall to this approach is that vessels with near-resolution or at-resolution radii are incorrectly sized when their closest non-vessel neighbor is connected by a voxel face or a pixel edge (Figure 2C), leading to oversized single-voxel/-pixel vessels (Figure 2D). This issue is not as apparent for non-vessel neighbors connected to voxel edges/corners or pixel corners (Figures S1D–S1F). As such, a simple half-unit correction is implemented along specific orientations to preserve small-segment radii measurements for non-vessel neighbors connected to voxel faces and pixel edges (Figures 2C–2F).

### VesselVio provides detailed feature reporting

Following the identification of branchpoints, endpoints, and centerline radii, individual vessel segments and their features can be isolated from vascular networks. Segment features extracted in the analysis pipeline include average length, radius, tortuosity, surface area, and volume. Next, this information is used to identify network features, including network volume and skeleton length, vessel segment partitioning, segment counts, and averages of segment features. Graph file exports from VesselVio enable users to independently identify relevant network characterization metrics, such as cohesion, network diameter, or clustering coefficient measurements (Hahn et al., 2019). Additionally, the centerline coordinate information extracted from the network are stored in the graph under a coordinates attribute, which can be accessed for custom geometric analyses. Lastly, by leveraging the same back-end analysis pipeline with an additional mesh-construction stage, VesselVio can render vascular datasets for visualization and inspection of results (Figure 1C).

### 3D dataset compatibility and utility testing

#### Analysis of annotated mouse cerebrovasculature imaged with LSM

**Figure 3:**
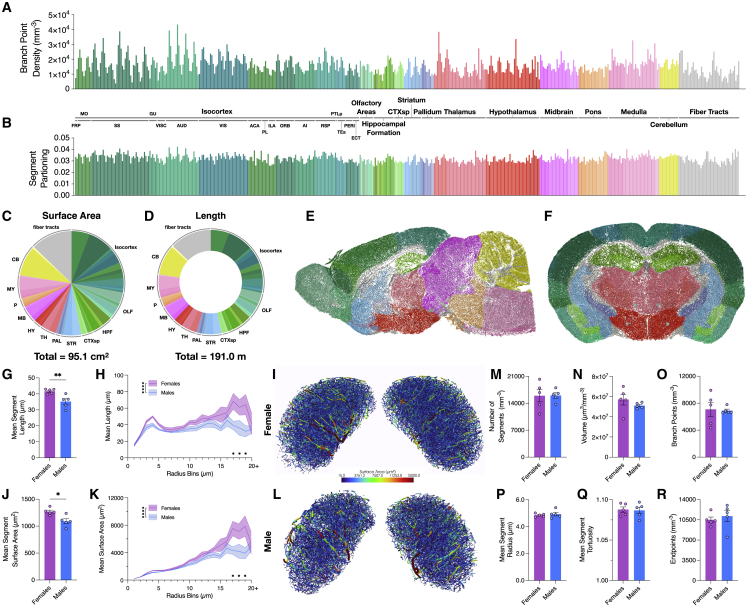
Analysis of BALB/c whole-brain vasculature datasets and CFW inferior colliculus (IC) vasculature segmentations highlight 3D analysis capabilities (A and B) Branchpoint (A) and vessel segment partitioning (B) across 483 subregions (n = 3). (C and D) The mean total vascular volume (C) and network length (D) of the BALB/c datasets. (E and F) Sagittal (E) and coronal (F) views of BALB/c cerebrovasculature colored with the corresponding Allen Brain Atlas CCFv3 annotations. (G and H) Sex difference in the mean segment length (G) and the mean length (H) of large diameter vessels in the IC (n = 5). (J and K) Sex difference in the mean segment surface area (J) and the mean surface area (K) of large diameter vessels in the IC. (I and L) Visualizations of the mean vessel surface area in the female and male IC. (M–R) Comparisons in the (M) number of segments, (N) network volume, (O) branchpoint density, (P) mean segment radius, (Q) mean segment tortuosity, and (R) endpoint density. Data are represented as mean ± SEM. (G, J, and M–R) Data analyzed using two-tailed Student’s *t* test. (H and K) Data analyzed using two-way ANOVA. Multiple comparisons were conducted using Sidak’s test. ∗p < 0.05, ∗∗p < 0.01, ∗∗∗∗p < 0.0001.

To demonstrate compatibility with 3D volumes, multiple datasets were analyzed. First, publicly available BALB/c mouse whole-brain vasculature datasets were acquired; these datasets were imaged with LSM (Todorov et al., 2020). To examine inter-regional network characteristics, 483 structures representative of the whole brain were selected using the Annotation Processing page (Figure S1C). The brains were subsequently loaded and analyzed (n = 3; Figures S2A–S2F). Branchpoint and segment density observations revealed connectivity variations among the major regions (Figures 3A and 3B). An average 95.1 cm^2^ of vascular surface area (Figure 3C) and an average 190.0 m of vasculature were observed in the brains (Figure 3D). Further, an average of 3.5 million branchpoints were identified across the brain (Table S1A). Network surface area and length were observed to be largely proportioned within the isocortex and fiber tracts (Figures 3C and 3D).

### Analysis of male and female mouse inferior colliculus segmentations imaged with μCT

Next, cerebrovascular resin casts of female and male CFW mice (n = 5) were created, as described previously (Quintana et al., 2019). Following cast creation, imaging, and volume reconstruction, the inferior colliculi (IC) were segmented and analyzed (Video S1; Video S2). Network feature analysis revealed two primary sex differences in these datasets: female mice had greater average segment length and segment surface area compared with males (Figures 3G and 3J), with peak differences occurring in the 17–19 μm vessel ranges (Figures 3H and 3K). Numerous other features were analyzed, but no sex differences were observed (Figures 3M–3R). Analysis runtimes of these datasets were compared with the VesSAP pipeline (Todorov et al., 2020). VesselVio analysis runtimes (32.2 ± 21.5 s) outperformed VesSAP runtimes (341.5 ± 78.9 s; Figure S2G). Furthermore, the runtime scaling of the pipeline was tested; processing speed was observed to scale linearly with voxel counts (Figure S2H).

Video S1. Mouse inferior colliculus vasculature manual segmentation, related to Figure 3

Video S2. Female mouse inferior colliculus vasculature visualization, related to Figure 3

### Compatibility testing with additional 3D datasets

Several other 3D vasculature datasets were also examined. First, an isotropic rodent lymph node microvascular network imaged with synchrotron μCT was visualized to reveal complex capillary networks (Video S3) (Jafarnejad et al., 2019). Next, the arterial and venous cerebrovasculature of an individual human brain from a public dataset was composited and visualized (Video S4) (Bernier et al., 2018). Finally, an anisotropic extraction of cerebrovasculature from C57 mice was examined (Video S5) (Di Giovanna et al., 2018).

Video S3. Mouse lymph node vasculature visualization, related to STAR Methods

Video S4. Human left-hemisphere cerebrovasculature visualization, related to STAR Methods

Video S5. Mouse cerebrovasculature visualization and application demonstration, related to STAR Methods

Together, the analysis and visualization of rodent cerebrovasculature, rodent lymph node, and human cerebrovasculature datasets demonstrate VesselVio’s compatibility with 3D datasets generated with various imaging techniques.

### Analysis of human 2D retinography datasets highlights disease-specific vascular phenotypes

**Figure 4:**
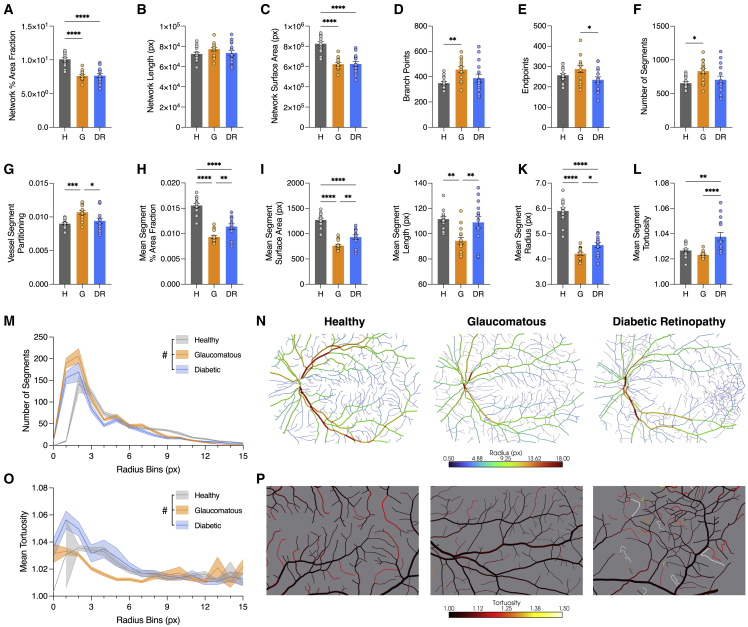
Analysis of images from the HRF database demonstrate compatibility with 2D datasets (A–G) Comparisons of network features for health (H), diabetic (D), glaucomatous patients (G; n = 15), including (A) vascular percent area fraction, (B) length, (C) surface area, (D) branch points, (E) endpoints, (F) segment counts, and (G) segment partitioning. (H–L) Averaged segment features, including (H) segment percent area fraction (PAF), (I) surface area, (J) length, (K) radius, and (L) tortuosity. (M) Distribution of segments across 0–15 px radius segment bins. (O) Mean tortuosity of segments in 0–15 px radius segment bins. Data are represented as mean ± SEM. (A–L) Data analyzed using a one-way ANOVA. (M and O) Data analyzed using a two-way ANOVA. Multiple comparisons were conducted using Sidak’s test. #, main effect of health condition, ∗p < 0.05, ∗∗p < 0.01, ∗∗∗p < 0.001, ∗∗∗∗p < 0.0001. (N) Visualization of the average vessel segment radius from representative images. (P) Visualization of the average vessel segment tortuosity from representative images.

To demonstrate the utility of VesselVio with 2D datasets, retinographs sourced from the HRF Image Database were analyzed and compared (Budai et al., 2013). This database contains high-resolution images of healthy control patients, patients with diabetic retinopathy, and patients with glaucomatous eyes. Analyses revealed several topological and segment-based differences among the groups. Relative to healthy controls, diabetic patients present with reductions in vessel-percentage-area fraction and surface area (Figures 4A and 4C), reduced average segment radius (Figure 4K), and increased vessel tortuosity (Figures 4L and 4O). Next, relative to healthy controls, glaucomatous retinas had reduced vascular surface area (Figures 4A and C), increased branchpoint counts (Figure 4D), increased segment counts (Figure 4F), and reduced average segment length (Figure 4J). Finally, the mean radius of segments was reduced in both groups compared with healthy controls, as well as the distribution of segments across small- and medium-radius bins (Figures 4K and 4M).

## Discussion

We developed an open-source application, VesselVio, for the analysis and visualization of segmented 2D and 3D vasculature datasets. Focus was centered on constructing an analysis pipeline capable of producing quantitative characterizations of numerous whole-network and small-scale segment features. Ground-truth result comparisons and three sets of analyses were conducted to demonstrate the accuracy of VesselVio for vasculature analysis.

The first two sets of analyses examined whole-brain vascular characteristics of BALB/c mice and sex-differences in the cerebrovasculature of female and male CFW mice. There are many sex differences in the cardiovascular system, including vascular tone (Boese et al., 2017), microcirculation (Huxley and Kemp, 2018), and blood-brain barrier function (Robison et al., 2019; Humphries et al., 2017). In the latter analysis, we sought to examine how the cerebrovascular network in a specific nucleus, the IC, may differ by sex. IC analyses revealed differences in the average length and surface area of segments, particularly large segments. The resulting whole-brain analyses provided continued insight into inter-regional differences in vascular density (Kirst et al., 2020; Ji et al., 2021). These results serve to demonstrate the 3D network analysis utility of VesselVio.

The third set of analyses examined retinal vascular networks of healthy controls, patients with diabetic retinopathy, and patients with glaucoma. Alterations in retinal microvascular networks are associated with vision loss (Carmeliet, 2005), and structural changes associated with excessive or reduced angiogenesis can also serve as indicators for underlying disease states (Lee et al., 2015; Selvam et al., 2018). Our analyses recapitulated previously reported reduced vessel area and diameter in glaucomatous eyes (Chan et al., 2017), as well as increased segment tortuosity in diabetic retinopathy eyes (Sasongko et al., 2011). Contrasting results in diabetic retinopathy eyes were noted in previous studies that reported increased vessel diameters (Bek, 2017; Klein et al., 2012), whereas we and others observed decreased diameters (Adhi et al., 2013; Fryczkowski et al., 1988). However, these differences may be due to the vessel type (the high-resolution fundus [HRF] vessels are not separated by arterial/venous hierarchy) or the vascular region analyzed. Although VesselVio is not intended to be used in any clinical diagnostic contexts, this analysis demonstrates the ability of this application to identify and characterize vascular network alterations in pathological conditions.

Future studies seeking to pinpoint detailed microvasculature alterations or broadly characterize regional vascular network features can employ the analytical and visualization components of VesselVio to obtain and enhance understanding of their results. In sum, VesselVio bridges the gap between modern vasculature imaging and analysis techniques by providing an optimized, open-source analysis pipeline and user-friendly application free for use.

### Limitations of the study

In common with most analytical programs, the functionality of VesselVio is limited by the quality or resolution of the images that are loaded into the program. For example, if anisotropic datasets are loaded without pre-smoothing filters, then skeletonization of these datasets can produce erroneous segments. To adjust for these errors, the option to prune small, connected end segments is included (Video S6), but this pruning is unbiased and could hinder some types of analyses, such as angiogenesis assessments.

Video S6. Segment processing demonstration, related to STAR Methods

Rather than analyzing whole-brain datasets at once without subregion selection (e.g., brainstem analysis), in this pipeline it is optimal to segment the brain into smaller regions prior to analysis (e.g., interbrain, midbrain, and hindbrain analysis). This is because region-based analysis occurs after region segmentation. A limitation of this approach is that region annotation prior to skeletonization can lead to boundary effects, where vessels that cross annotation boundaries become disconnected during graph construction. The most notable effect of this disconnection would be altered endpoint counts. This approach was selected because of the offered speed optimizations and drastic memory usage reductions, as specific research questions often analyze subregional vasculature rather than whole-brain vasculature. In contrast, because of these boundary effects, it may be argued that VesselVio is most suitable for the analysis of small- to medium-sized datasets where subregion analysis is not necessary. Future approaches may seek to find a balance between this pipeline and other headless open-source pipelines, such as TubeMap (Kirst et al., 2020), that avoid boundary effects by requiring greater memory availability.

The presence of spurious branchpoints is unavoidable in the process of the implemented skeletonization algorithm and 26-connectivity graph construction. When holes or imaging artifacts are present in datasets, the skeletonization algorithm can also produce erroneous centerlines. VesselVio takes an automated approach to correct these spurious branchpoints and erroneous centerlines. A limitation to the automated branchpoint-filtering algorithms may mean that in some instances, the automated correction of spurious branchpoints or erroneous centerlines may be less optimal than manual corrections. However, given that there can be hundreds to hundreds of thousands of spurious branchpoints in skeletonized datasets, manual corrections are not feasible. Future contributions may seek to implement user-guided graph construction that can match the efficiency of our automated approach.

Because VesselVio requires that binarized datasets be loaded for analysis, our radius calculations are based on the binary volume and its centerline. Though our modified Euclidean distance transform (mEDT) radius calculations enable more detailed radius assessments than the traditional EDT calculations, this radius calculation method is likely not as effective as another recent approach that uses vessel-filled datasets, which are created with corrosion casting or fluorescent gel perfusion techniques. This approach adjusts uncentered centerlines and calculates the radius of centerline voxels based on an iterative assessment of vessel fluorescence intensity and angle (Ji et al., 2021). As such, future contributions to this application may seek to support radius calculations for vessel-filled datasets that are based on this described approach (Ji et al., 2021).

Finally, because VesselVio is a graphical user interface (GUI)-based application primarily intended for standalone downloads, bundling the application for distribution acts as a limitation to speed. Bundling leads to slower performance in comparison to the non-bundled terminal-based version of our application or other headless analysis pipelines. Although functionality is equivalent between the bundled application and the application built from terminals at runtime, terminal builds offer more efficiency on startup and analysis. Because of this, single-line executable usage of the app is detailed in our documentation.

## STAR★Methods

### Key resources table

**Table:** 

REAGENT or RESOURCE	SOURCE	IDENTIFIER
**Chemicals, peptides, and recombinant proteins**		

Heparin Sulfate	McKesson Corporation	63739-931-28
Paraformaldehyde	Acros Organics	416780030
PU4ii Resin and Hardener	VasQTec	NA
Methyl Ethyl Ketone	Fisher Chemical	M209
Formic Acid	VWR International	BDH4554
Potassium Hydroxide	VWR International	BDH7622
Phosphate Buffered Saline	Gibco	10010-023
Osmium Tetroxide	Sigma Aldrich	75632
Isoflurane	VetOne	502017

**Deposited data**		

Mouse Inferior Colliculus Vasculature Datasets	Harvard Dataverse	https://doi.org/10.7910/DVN/PCXU6D
Synthetic Vasculature Datasets	Harvard Dataverse	https://doi.org/10.7910/DVN/TIOFR2
Human Retinal Fundus Image Database	Budai et al., 2013	https://www5.cs.fau.de/research/data/fundus-images/
CD1 Mouse Whole-Brain Vasculature Datasets	Todorov et al., 2020	https://github.com/vessap/vessap

**Experimental models: Organisms/strains**		

Female and Male CFW Mice	Charles River Laboratories	#024

**Software and algorithms**		

microCT 3D.SUITE Software	Bruker	NA
Prism 9.3.0	GraphPad	https://www.graphpad.com/scientific-software/prism/
ImageJ	Schneider et al., 2012	https://imagej.nih.gov/ij/
Python 3.8	Python Software Foundation	https://www.python.org
Adult Mouse Brain Atlas (p56, CCFv3)	Allen Institute	http://api.brain-map.org/api/v2/structure_graph_download/1.json
QuickNII v1	Puchades et al., 2019	https://www.nitrc.org/projects/quicknii
VesselVio Source Code	This paper	https://doi.org/10.5281/zenodo.6147198 and https://github.com/JacobBumgarner/VesselVio
VesselVio Application Downloads and Terminal-Build Instructions	This paper	https://jacobbumgarner.github.io/VesselVio/ and https://sourceforge.net/projects/vesselvio/

**Other**		

Teklad Global 18% Protein Rodent Chow	Teklad	2018
Skyscan μCT Scanner	Bruker	1272

### Resource availability

#### Lead contact

Further information and requests for code, datasets, or other resources used in this study should be directed to and will be fulfilled by the lead contact, Jacob Bumgarner (jrbumgarner@mix.wvu.edu).

#### Materials availability

This study did not generate new unique reagents.

### Experimental model and subject details

#### Animals

All experiments were approved by the West Virginia University Institutional Animal Care and Use Committee, and animals were maintained in accordance with NIH Animal Welfare guidelines. Adult female and male CFW mice (7- to 8-weeks of age; strain #024; Charles River Laboratories, USA) were obtained and maintained under 14:10 light-dark cycles (150 ± 25:0 lux light:dark; lights on from 0500-1900 h). Following arrival, animals were given 1 week to acclimate to vivarium conditions before tissue collection. Food (2018 Teklad; Envigo, USA) and reverse osmosis water were provided *ad libitum* throughout the entire duration of the experiment.

### Method details

#### Vascular corrosion casting and μCT imaging

Vascular casts of mouse brains were created using a resin corrosion casting method described previously (Quintana et al., 2019). Perfusions occurred in the light phase between 1200-1600 h. Prior to perfusion, mice were injected i.p. with 25 U of heparin (63739-931-28; McKesson Corporation, USA) in 250 μL of saline and then deeply anesthetized with an isoflurane (4% induction, 1.5% maintenance) and 0.4 L/min oxygen flow mixture. Following confirmation of complete anesthetization with a pedal withdrawal reflex test, mice were transcardially perfused at a flow rate of 4 mL/min first with 15 mL of 25 U/mL heparin in saline, followed by 15 mL of 4% paraformaldehyde (#416780030; Acros Organics, Belgium) in saline with a pH of 7.4, followed lastly with PU4ii resin (VasQTec, Switzerland) formulated exactly as directed by the manufacturer with methyl ethyl ketone dilution (M209, Fisher Chemical, USA). Five days after perfusion, the craniums were decalcified with a 12-h wash of 5% formic acid (BDH4554; VWR International, USA), the brains were dissected, and remaining tissue was removed from the casts with two 12-h washes of 7.5% KOH (BDH7622; VWR International, USA) at 50°C. Casts were then rinsed with three 1-hour Milli-Q water washes, and the cleaned casts were osmicated in a 1% solution of osmium tetroxide (#75632; Sigma Aldrich, USA) for 12-hours to allow for optimal x-ray diffraction during μCT scans. Casts were imaged on a SkyScan 1272 (Bruker, USA) at 50 kV/ 200 μA with 360° rotations in step sizes of 0.17°, no filter, 900 ms frame exposures, and 4 frame averages/step to produce an isotropic voxel resolution of 2.7 μm^3^. Scan parameters were determined based on the manufacturer’s guidance to achieve optimal x-ray transmission through the sample. Scans were then reconstructed using NRecon (Bruker) with beam hardening corrections at 15%, ring artefacts reduction at 3, smoothing at 0, custom alignment compensations set for each sample, and 0.02-0.40 dynamic image ranges. Following reconstruction, the volumes were resliced coronally for inferior colliculi segmentations. Using interpolated polygonal tracing with CTAn (Bruker), inferior colliculi were manually segmented from bregma −4.9 to −5.4 using the lobule 2 of the cerebellar vermis as a landmark for bregma −4.9 (Video S1) (Paxinos and Franklin, 2019). Scans, reconstructions, reslicing, and inferior colliculi segmentations were all conducted using the Bruker SkyScan analysis software suite. The resulting segmentatinos were analyzed at a 2.7 μm^3^ isotropic resolution with a 10 μm isolated segment filter length and a 5μm endpoint segment prune length (Table S1B).

#### Dataset details

##### BalbC mouse brain vasculature datasets

The BalbC whole-brain vasculature datasets were sourced from the VesSAP repository (Todorov et al., 2020). To analyze the whole brain datasets, 483 regions were selected using the Annotation Processing page. The downloaded brains were loaded directly into the program without alteration and were analyzed at a 3 μm^3^ isotropic resolution with a 10 μm isolated segment filter length and a 5μm endpoint segment prune length (Table S1A).

##### HRF dataset

The HRF image dataset was downloaded directly from the HRF Image Database (Budai et al., 2013). The images were loaded directly into the program and analyzed with a 10 px isolated segment filter length and a 5 px endpoint segment prune length (Table S1C).

##### Synthetic dataset

Twenty synthetic vasculature datasets with corresponding branch point label datasets were downloaded and holes in the vasculature were manually filled (Tetteh et al., 2020). The datasets were then loaded into the program and analyzed with a 10 voxel isolated segment filter length and a 5.5 voxel endpoint segment prune length. Branch point labels from the labeled datasets were quantified by skeletonizing the spheres around the branch points, labeling the components, and counting the components (Table S1E).

#### VesselVio pipeline

##### Dataset input and processing preparation

Images are loaded into VesselVio using the Simple-ITK image reader or nibabel, depending on the filetype (Lowekamp et al., 2013; Brett et al., 2020). It is important to note that VesselVio is only compatible with vascular datasets that have been pre-segmented. To ensure that datasets loaded into the program are prepared appropriately for subsequent analysis, all loaded volumes are binarized and prepared as n-dimensional contiguous arrays of 0-value background and 1-value foreground elements. All array processing in VesselVio is conducted using NumPy and Numba (Harris et al., 2020; Lam et al., 2015).

##### Volume skeletonization and centerline extraction

VesselVio employs a custom implementation of a widely used medial axis parallel thinning algorithm to locate vessel centerlines from 2D and 3D datasets (Lee et al., 1994), with speed optimizations and parallel processing enabled by Numba. This algorithm was selected because it produces comparatively few erroneous branch point extensions, particularly when used with high-resolution datasets. This algorithm is also capable of thinning 2D and 3D datasets, making it optimal for this pipeline. Following skeletonization, (n, 3) or (n, 2) arrays are created containing Cartesian coordinate information of the location of all vessel centerlines.

##### Radii calculations

Before the creation of the undirected graph, vessel centerline radii measurements are conducted. Previous publications focused on voxel/pixel vasculature analysis have utilized Euclidean distance transforms (EDT) on segmented vasculature images to find centerline radii (Tsai et al., 2009; Todorov et al., 2020). These blanketed EDT methods find the Euclidean distance (ED) from the centerline point to the center of the nearest non-vessel neighbor point. However, an apparent unconsidered flaw in this traditional method is that finding the ED to the nearest non-vessel neighbor can overestimate the radius of the vessel if its nearest non-vessel neighbors are 6-connected in 3D space (N_6_(v)) or 4-connected in 2D space (N_4_(p)) (Table S1D). For example, given an isotropic voxel resolution of 1 μm^3^, a straight vessel along the X-axis that is 1 voxel thick should have a putative radius of 0.5 μm (Figure S1D). However, the traditional EDT method will record this vessel as having a radius of 1 μm^3^, doubling the apparent diameter. This is because ED measurements are calculated between the coordinates of the center of the centerline voxel and the *center* of its nearest non-vessel neighbor. Radii inflations in traditional EDT measurements are less pronounced as vessels become larger, but small-diameter vessels can be binned incorrectly using this blanketed technique.

To avoid this overestimation of vessel size, distance calculations can be made to the *face* of N_6_(v) or the *edge* of N_4_(p) non-vessel neighbors. To achieve this, a modified EDT (mEDT) calculation was implemented with 0.5-unit corrections for all N_6_(v) or N_4_(p) non-vessel neighbors (Figures S1D–S1F). When the nearest non-vessel neighbors are located beyond the immediate N_26_(v) or N_8_(p) space, the mEDT is applied to points that are connected to the centerline point only via face-to-face steps in 3D space or edge-to-edge steps in 2D space (i.e., not to diagonally connected points). In other words, given the phenotypical tubular shape of vessels, the mEDT approach is not applied to non-vessel neighbors of the sets N_26_(v) - N_6_(v) and N_8_(p) - N_4_(p) or non-vessel points beyond N_26_(v) or N_8_(p) neighborhoods that are situated diagonally to the centerline point (Figures S1D–S1F).

In previous publications, EDTs are applied to the entire image, and the centerline values are then extracted. However, this process is computationally expensive and leads to difficulty with making the intended 1D corrections. As such, the mEDT method is only applied to centerline points, rather than the entire image. Points in the previously constructed centerline coordinate arrays are used as seeds for the placement of expanding boxes that search centerline point neighborhoods for non-vessel neighbors. This box expands around a centerline point until at least four non-vessel neighbors in the binarized volume are identified, rather than a single neighbor as with previous implementations. Then, rather than directly computing the ED (square root of sum of squared deltas) between the centerline point and neighbor (1), the absolute deltas of the centerline and neighbor coordinates are computed and loaded into a lookup table with precomputed mEDT values (2). This lookup table is constructed based on the input resolution to provide the half unit corrections along the described orientations (e.g., an XYZ delta array of [0,0,5] returns a 4.5 voxel distance).

$$\left(1\right)d_{\text{radius}}\left(c,b\right)=\sqrt{\sum_{i=1}^{n}{\left(c_{i}-b_{i}\right)}^{2}}\;\left(2\right)d_{\text{radius}}=mEDT_{LUT}\left[|c_{x}-b_{x}|,\;|c_{y}-b_{y}|,\;|c_{z}-b_{z}|\right]$$

After the distance for each non-vessel neighbor is calculated, the four lowest values are averaged, and this average is defined as the radius for the centerline point. Four values are averaged to improve the accuracy of radii measurements and account for potential small vessel surface divots, bubbles, or imaging artifacts common in various vascular preparation techniques, such as corrosion casting. This process is parallelly repeated for all centerline points.

##### Graph construction

Following radii measurements, undirected graphs are constructed to represent the vasculature skeleton. Graph creation and processing in VesselVio uses the Python igraph package (Csardi and Nepusz, 2006). To construct the vascular graphs, the number of centerline points is identified, and an equal number of isolated vertices are added to the graph. At this point, 2D images are padded as 3D arrays. Centerline points are assigned an index based on their order of appearance in the point coordinate array. Each vertex is given a coordinate and radius attribute based on its corresponding point values. Next, the [x_max_, y_max_, z_max_] oriented 13-connectivity neighborhood of all centerline points is scanned to identify N_26_(v) neighbors. Upon neighbor identification, an edge is created between the vertices. Unidirectional scanning in the edge identification process prevents parallel edges from being created (Kirst et al., 2020).

##### Clique cluster processing

Branch points in the constructed graphs are identified based on degrees of connectivity; if a vertex has a degree greater than two, it is defined as a branch point (Todorov et al., 2020; Arganda-Carreras et al., 2010). Because the graphs are constructed based on 26-connecitivity, this identification method leads to an artificial inflation of identified branch points. This is because at individual branch point junctions, multiple vertices can have > 2 neighbors (Figure 2A). In graphical space, these falsely identified branch points form small all-to-all connected loops, or cliques; clusters of these cliques are also common occurrences.

To correct spuriously labeled branch points, a two-pass filtering algorithm was developed that led to a correction accuracy of 97.4% (Figure 2B). This approach resolves clique clusters that are categorized into three classes. In the first pass, class 1 clusters are eliminated; these clusters were observed to be the most common type of cluster present in the examined datasets (Table S1F). Class 1 clusters are identified by isolating maximal cliques with three/four vertices from the main graph. Vertices in these cliques are then weighted based on their radius and the radius of their neighbors in the main graph (Figure S1G). The connection between the lowest weighted candidates in the clique is then removed, thereby eliminating three-vertex cliques and simplifying four-vertex cliques.

In the second pass, class 2 and 3 clique clusters are corrected. These classes are large clusters of cliques that arise due to binarization, vessel filling, or skeletonization errors. Class 2 clique clusters contain < 50 vertices, whereas class 3 clusters contain ≥ 50 vertices. Class 2 clusters are eliminated by finding the mean radius of the vertices, identifying external projections, and creating a new vertex with this mean radius that connects to the identified projection targets (Figure S1H). The class 2 filter also resolves the previously simplified four-vertex cliques. Class 3 clusters are eliminated using a sliding window approach that scans over the cluster along its longest axis. In each window, the same algorithm for class 2 is applied, where a new single vertex is created with the mean radii of the original window vertices and preserved external projections. The new vertices created along the sliding window are then connected (Figure S1I).

##### Isolated and endpoint segment processing

Following graph creation, n != 2 degree vertices are filtered from the graph. The remaining components are then scanned, and isolated segments and endpoint segments shorter than user-defined lengths are pruned (Video S6). Following the removal of isolated segments from the graph, voxels/pixels are also removed from the corresponding segment in the volume for visualization purposes.

##### Feature extraction

All individual segment characteristics and whole-network features are extracted from the constructed graph. Branch point and endpoint counts are determined by the number of n > 2 and n = 1 degree vertices in the graph, respectively. Segments are identified in the graph by filtering n > 2 degree vertices and then sorting through the remaining individual components. After each segment is identified, the mean, minimum, maximum, and standard deviation of the segment radius is calculated. Because calculating segment length based on vertex-vertex edges produces paths that are irregular to the vessel surface, a smoother path is constructed by creating B-splines of varying degrees from the coordinates of the original segment vertices using the geomdl package (Bingol and Krishnamurthy, 2019). The length along the spline is then approximated using ED calculations between a defined number of points along the spline identified using the *Cox-De Boor* algorithm (Bingol and Krishnamurthy, 2019). Then, tortuosity measurements are created by finding the arc-cord ratio of the segment (segment length divided by the ED between the start and end points). Finally, the lateral surface area and volume of the segment are found using the mean radius and segment length. Averages of segments are then identified and automatically binned for ease of analysis. Segment partitioning is also calculated by dividing the number of segments by the total network length (Corliss et al., 2019).

##### Annotated volume processing

Datasets can be loaded alongside annotated volumes for subregion analysis. First, a JSON annotation processing file is created on the Annotation Processing page. Users can select specific regions of interest from several pre-loaded Allen Brain Atlas annotation trees, including the CCFv3 p56 mouse brain tree (Wang et al., 2020), can load their own annotation trees, or can create individual ROI identifiers for custom annotations, such as those created with ITK-Snap. Then, this annotation file is loaded alongside the binarized vascular volume and the annotated volume dataset for analysis/visualization. Volumes with integer/float id-based annotations must be loaded as NIfTI files for annotation analysis. Separately, RGB-based annotations (e.g., .png series of RGB annotations) can be loaded. RGB annotation testing was conducted using QuickNII (Puchades et al., 2019).

The backend pipeline analyzes annotation regions (ROIs) in bins of 254 annotations. During each bin iteration, the selected ROIs are assigned values from 1-255, and the IDs from the identified ROI regions are casted onto the correlating voxels in the vasculature dataset to create a labeled vasculature dataset. This labeled dataset is then temporarily cached on the disk as a NumPy file for memory-mapped dataset access to prevent excessive working memory requirements. During the ROI segmentation process, the volume of each ROI is calculated and stored. At the stage of analyzing each ROI, the individual ROI-associated vasculature is segmented from the temporary labeled dataset for subsequent skeletonization and analysis. Following the analysis of each ROI, the constructed graph is added to a single main graph with preserved spatial and connectivity information. This main graph can then be exported for custom analyses. However, because most graph formats do not allow list/array attributes, the coordinates of the path of segments are eliminated during the process of reducing the centerline-based graph (vertices represent centerlines, edges represent centerline connections) to a branch-point based graph (vertices represent branch points, edges represent segments). Instead, this geometric segment path information can be retrieved through custom modifications, as the segment coordinates are stored as an edge attribute that can be extracted or modified prior to saving the graph.

##### Mesh visualization and application interface

To construct meshes for visualization, we leverage the high-level Python package PyVista (Sullivan and Kaszynski, 2019) that wraps The Visualization Toolkit. We create individual polydata datasets from our segment splines, apply tube filters to create centerline and scaled network meshes, and assign each tube a radius, length, tortuosity, surface area, and volume scalar for visualization. All scalars and scaled segment sizes are based on the mean of segment features. These segments are combined into an undirected grid for surface extraction and subsequent rendering. Additional branch point and endpoint meshes are created. Then, original (voxel/pixel based) and smoothed surface meshes (marching cubes based-based) are created from the filtered input volume so researchers can visually validate the output features from the program by comparing simple/scaled networks to the original/smoothed meshes. Lastly, construction of the front-end application for VesselVio was accomplished with PyQt5 under GNU GPLv3 licensing (Figures S1A–S1C).

##### Computational resources

VesselVio was developed with Python 3.8 and was tested locally on a 2019 16″ MacBook Pro (2.6 GHz 6-Core Intel Core i7, Intel UHD Graphics 630 16GB) and a Windows 10 computer with dual 3.39 GHz Intel Xeon Gold 6128 processors, 128 GB of Sk Hyinx Ddr4 Sdram Memory Module RAM, and dual 8GB NVIDIA Quadro P4000 graphics cards.

### Quantification and statistical analysis

All statistical analyses were conducted using Prism 9 (GraphPad; USA). Synthetic vasculature results were analyzed using one-way ANOVA tests and repeated-measures two-way ANOVA tests. HRF datasets were analyzed using one-way ANOVA tests. Mice IC data were analyzed using two-tailed student’s *t* tests. Distributions of segment counts per radii bin, average segment length per radii bin, and segment tortuosity per radii bin were all analyzed using two-way ANOVA tests. Following main effects observations in one- or two-way ANOVA tests, multiple comparisons were made using Sidak’s test. P-values below 0.05 were considered statistically significant.

## Data Availability

• The synthetic vasculature and inferior colliculus vasculature datasets have been deposited at the Harvard DataVerse Repository and are publicly available as of the date of publication. DOIs are listed in the key resources table. The BalbC whole-brain vascular datasets can be found via the VesSAP respository (https://github.com/vessap/vessap). The HRF dataset can be downloaded from the HRF Image Database (https://www5.cs.fau.de/research/data/fundus-images/). • All original code has been deposited at https://zenodo.org and is publicly available as of the date of publication. DOIs are listed in the key resources table. Downloads for Windows and MacOS as well as the source code can be found on the VesselVio GitHub repository (https://github.com/JacobBumgarner/VesselVio). • Any additional information required to reanalyze the data reported in this paper is available from the lead contact upon request.

## Supplemental information

Document S1. Figures S1 and S2

Table S1. Data from the analysis of mouse whole-brain, mouse inferior colliculus, human retinography, and synthetic vasculature datasets, related to Figures 2, 3, and 4

Document S2. Article plus supplemental information
