# Supplementary material for: Open-source analysis and visualization of segmented vasculature datasets with VesselVio
Source: Cell Rep Methods. 2022 Mar 23;2(4):100189. doi: 10.1016/j.crmeth.2022.100189 (PMC9046271; doi:10.1016/j.crmeth.2022.100189)
Supplement: Document S1. Figures S1 and S2 [file mmc1.pdf]

Cell Reports Methods, Volume *2*

Supplemental information

Open-source analysis and visualization of segmented vasculature datasets with VesselVio

Jacob R. Bumgarner and Randy J. Nelson

Table of Contents:

1. Supplemental Figures 1-2
2. Supplemental References

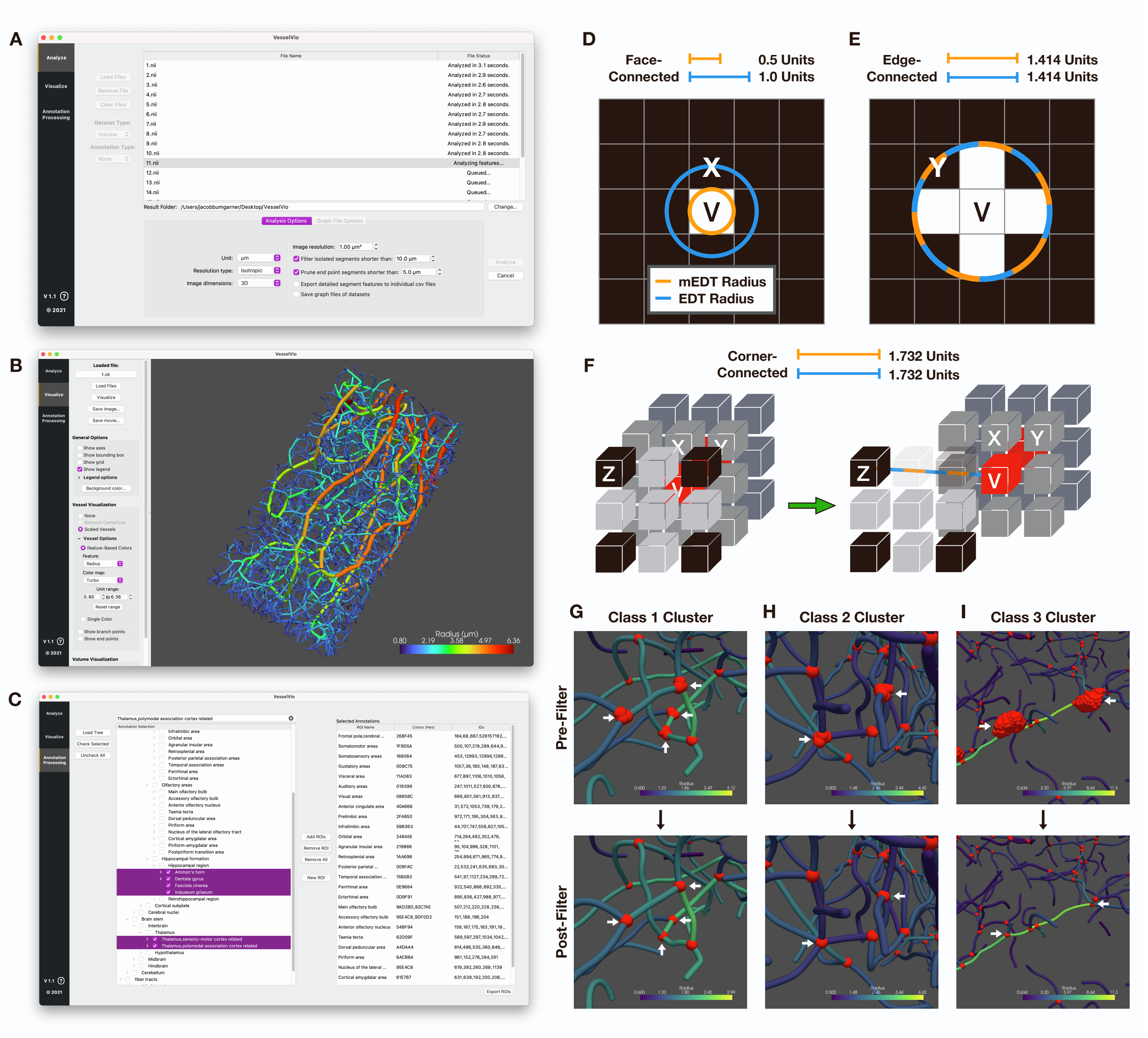

Figure S1 – VesselVio application interface; Euclidean distance transform (EDT) vs. modified EDT (mEDT) radius calculations; and Clique cluster filtering examples, related to Figures 1 and 2. (A) The analysis page, where binarized volumes and preconstructed graphs can be loaded and analyzed alongside dataset annotations. (B) The visualization page for observation of vascular networks. Meshes can be colored by segment radius, length, tortuosity, surface area, volume, or percent area fraction features with various color themes, depending on analysis parameters. Networks can also be visualized alongside original voxel/pixels or smoothed volume meshes. (C) The annotation processing page, where several default brain annotation trees from the Allen Brain Institute can be loaded for region specific vascular analysis. Custom trees can also be loaded for other species or structures in addition to custom individual regions of interest. (D) mEDT distance calculations from the centerline point [V] to nearest non-vessel neighbors connected by voxel faces or pixel edges [X] have 0.5 unit corrections. (E and F) mEDT distances to pixel corner-connected/voxel edge-connected [Y] as well as voxel corner-connected [Z] non-vessel neighbors are calculated in the same manner. (G, H, I) Example filtering results for class 1-3 clique clusters.

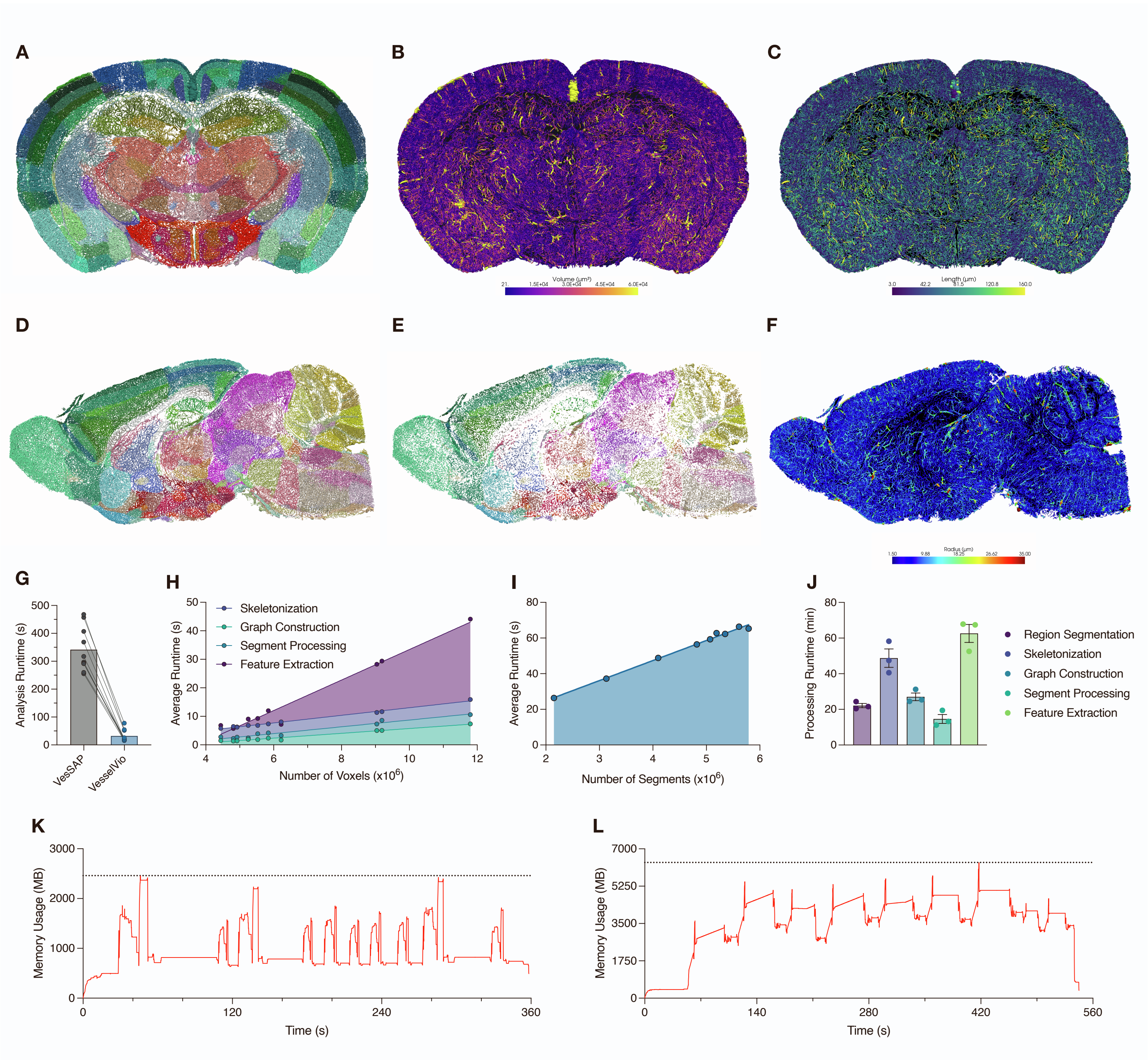

Figure S2 – Coronal and sagittal slice views of a BalbC cerebrovasculature dataset; VesselVio processing speeds and memory usage profiles, related to Figure 3. (A-C) A coronal slice of cerebrovasculature showing (A) colorized annotations, (B) segment volume scalars, and (C) segment length scalars. (D-F) a sagittal slice of cerebrovasculature showing (D) colorized annotations, (E) vessel branch point density colorized with region annotations, and (F) segment radius scalars. (G) VesselVio vs. VesSAP analysis speeds of the ten inferior colliculus datasets (*n*=10; 3 speed trials per point). (H) Runtime scaling of the individual steps of the analysis pipeline for the ten inferior colliculus datasets (3 speed trials per point). (I) Linear runtime scaling (n=9; 3 speed trials per point) of the analysis of nine whole-brain pre-constructed graphs generated by Paetzold *et al.* 2021, *arXiv*. (J) Analysis runtimes for individual components of the analysis pipeline during the analysis of the three BalbC whole-brain datasets generated by Todorov *et al.* 2020, *Nature Methods*. (K) Memory usage profile of the analysis of the ten inferior colliculus datasets. (L) Memory usage profile of the analysis of the nine whole-brain pre-constructed graphs.

Supplemental References

Paetzold, J. C., McGinnis, J., Shit, S., Ezhov, I., Büschl, P., Prabhakar, C., Todorov, M. I., Sekuboyina, A., Kaissis, G., and Ertürk, A. (2021). Whole brain vessel graphs: A dataset and benchmark for graph learning and neuroscience (vesselgraph). arXiv preprint arXiv:2108.13233

Todorov, M. I., Paetzold, J. C., Schoppe, O., Tetteh, G., Shit, S., Efremov, V., Todorov-Völgyi, K., Düring, M., Dichgans, M., and Piraud, M. (2020). Machine learning analysis of whole mouse brain vasculature. Nat. Methods *17*, 442-449.
